# A Computational Analysis of ATP Binding of SV40 Large Tumor Antigen Helicase Motor

**DOI:** 10.1371/journal.pcbi.1000514

**Published:** 2009-09-25

**Authors:** Yemin Shi, Hanbin Liu, Dahai Gai, Jianpeng Ma, Xiaojiang S. Chen

**Affiliations:** 1 Molecular and Computational Biology, University of Southern California, Los Angeles, California, United States of America; 2 Chemistry Department, University of Southern California, Los Angeles, California, United States of America; 3 Verna and Marrs McLean Department of Biochemistry and Molecular Biology, Baylor College of Medicine, Houston, Texas, United States of America; 4 Department of Bioengineering, Rice University, Houston, Texas, United States of America; The University of Texas at Austin, United States of America

## Abstract

Simian Virus 40 Large Tumor Antigen (LTag) is an efficient helicase motor that unwinds and translocates DNA. The DNA unwinding and translocation of LTag is powered by ATP binding and hydrolysis at the nucleotide pocket between two adjacent subunits of an LTag hexamer. Based on the set of high-resolution hexameric structures of LTag helicase in different nucleotide binding states, we simulated a conformational transition pathway of the ATP binding process using the targeted molecular dynamics method and calculated the corresponding energy profile using the linear response approximation (LRA) version of the semi-macroscopic Protein Dipoles Langevin Dipoles method (PDLD/S). The simulation results suggest a three-step process for the ATP binding from the initial interaction to the final tight binding at the nucleotide pocket, in which ATP is eventually “locked” by three pairs of charge-charge interactions across the pocket. Such a “cross-locking” ATP binding process is similar to the binding zipper model reported for the F1-ATPase hexameric motor. The simulation also shows a transition mechanism of Mg^2+^ coordination to form the Mg-ATP complex during ATP binding, which is accompanied by the large conformational changes of LTag. This simulation study of the ATP binding process to an LTag and the accompanying conformational changes in the context of a hexamer leads to a refined cooperative iris model that has been proposed previously.

## Author Summary

The Large Tumor antigen (LTag) encoded by Simian Virus 40 (SV40) is a marvelous molecule that is not only a viral oncogene, but also an efficient molecular machine as a helicase that unwinds double helix DNA for genome replication, an essential process in all living organisms. LTag hexameric helicase uses the energy of ATP to power its conformational switch for DNA unwinding. Understanding how the LTag conformational switch is coupled to the energy from ATP usage by LTag to do the mechanical work of unwinding DNA is of great interest to biologists, and yet remains to be established. Based on our previous high-resolution structures of LTag helicase in different conformational states, we simulated an LTag conformational transition pathway in the ATP binding process using the targeted molecular dynamics method. Our simulation results suggest a three-step process for the ATP binding to the nucleotide pocket, in which ATP is eventually “locked” into the pocket by three pairs of “locker” interactions. We have also quantitatively evaluated the energy profile of ATP binding using a special computational simulation technique. Additionally, our simulation study of ATP binding by LTag and the accompanying conformational switches in the context of a hexamer leads to a refined cooperative iris model that may be used for DNA unwinding.

## Introduction

Helicases are a family of ATPase motors that couple the energy of ATP binding and hydrolysis to conformation changes, which in turn is coupled to the unwinding and translocation of DNA [1]. Simian Virus 40 (SV40) large tumor antigen (LTag) is an efficient hexameric helicase that belongs to the helicase superfamily III, as well as the AAA+ protein family.

The high resolution structures of LTag hexameric helicase in different nucleotide binding states have been previously reported [2],[3], including the Apo, the ATP-bound and the ADP-bound states. These three structures reveal an iris-like motion of the hexamer helicase during the drastic conformational switches that are triggered by ATP binding and hydrolysis. Accompanying the iris-motion of the LTag hexamer is the longitudinal movements of the six β-hairpins along the central channel. Despite the advancement in LTag helicase studies mentioned above, the detailed paths for these conformational switches and the corresponding energetics associated with the ATP binding process are unknown, which can be simulated by a computational approach using molecular dynamics and targeted molecular dynamics.

Molecular dynamics (MD) propagates the molecular system under the laws of classic mechanics [4],[5], and is suitable for studying conformational changes. However, the current computational capability restricts the size (molecular weight) of the studied system and the time scale of MD simulation. For the studies of larger and more complex systems, targeted molecular dynamics (TMD) has been used to accelerate the simulation, which adopts an additional holonomic constraint on the physical potential to reduce the root mean square deviation between the current structure and the final (targeted) structure [6]. TMD is suitable to calculate the transition pathways between two known protein conformations. The combination of MD and TMD methods have been widely applied to the dynamics studies of various systems, such as the Ras p21 in the signal transition pathway [7], F1-ATPase system [8],[9], the GroEL complex [10], and the human a-7 nAChR receptor [11]. Here we adopted TMD to calculate the whole transition pathway and used MD to simulate the accurate conformational change in certain key time slots.

In order to understand the energetic aspects of ATP triggered conformational changes of LTag hexameric helicase, we simulated the ATP binding process of LTag and the associated conformational changes. We first built an Apo state with six ATPs placed 20 Å away from the binding pocket of the original Apo structure. Then we used the TMD approach to calculate the transition pathway from the Apo state to the ATP bound state and examined the ATP binding process that powered this conformational transition. The results suggest an ATP molecule goes through a three-step process before being “locked” inside the nucleotide pocket. Meanwhile, the configurations of the binding pocket along the ATP binding pathway were evaluated by using the linear response approximation (LRA) version of the semi-macroscopic protein dipoles langevin dipoles method (PDLD/S), a method that is capable of evaluating binding free energies significantly faster than the microscopic methods with comparable accuracy [12]. In addition, the simulation results of the conformational transition reveal a refined pathway for the cooperative iris-like movement of LTag hexamer helicase previously observed in crystal structures.

## Results

### Overall structure of SV40 LTag helicase domain

**Figure 1:**
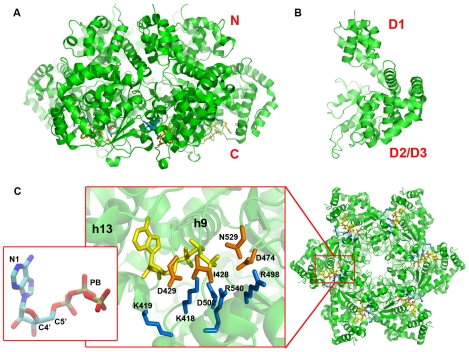
The structural features of LTag helicase and its nucleotide pocket. **(A)** The side view of the hexamer structure of LTag helicase. **(B)** The side view of LTag monomeric structure. **(C)** The structure of the LTag helicase binding pocket viewing from C-terminal end (bottom). The *cis*-residues are in copper and the *trans*-residues are in blue. The ATPs are painted in yellow in the middle figure. The ATP is colored by element type in the left mini-view. The nitrogen, carbon, oxygen and phosphate atoms are painted in blue, cyan read and gold. The N1-C4′-C5′-PB dihedral angle and the N1-C4′-PB bending angle are used to represent the conformational change in the ATP binding procedure.

There are three high-resolution LTag hexameric helicase structures corresponding to different nucleotide bound states [2], namely, the Apo state (PDB ID 1svo), the ATP bound state (PDB ID 1svm) and ADP bound state (PDB ID 1svl). The hexameric helicase structure reveals two stacked hexamer rings with a central channel (Fig. 1 (A)).

Each LTag subunit of the hexamer contains three structural domains, D1, D2 and D3 (Fig. 1 (B)). D1 is the N terminal Zn domain essential for LTag hexamerization [2],[13]. D2 is a typical AAA+ domain with Walker A or p-loop and Walker B motifs, which is important for ATP binding [2]. D3 is composed mostly of long helices, which is sequentially interrupted by D2 roughly in the middle of D3, while D1 is structurally well separated from D2/D3 (Fig. 1 (C)).

The binding pocket is located at the interface between two adjacent monomers. The monomer with the P-loop at a given interface is named *cis*-monomer, and the other monomer forming the interface is named *trans*-monomer. For ATP to bind to the nucleotide pocket, the only possible route is through the opening between the two neighboring monomers (or subunits) from the C-terminal end (bottom). The binding pocket residues on the *cis*-monomers can be divided into two groups, the I428, D429, K432, T433, T434 on the P-loop, and the N529, D474 on the Sensor I motif (Fig. 1 (C)). The binding pocket residues on the *trans*-monomers include the arginine finger tR540 (t designates *trans*) and lysine finger tK418, and residues tR540, tD502 and tR498. The ATP interacts with the *cis*-residues and *trans*-residues mainly through its phosphate group and the ribose. The adenosine group inserts into the hydrophobic pocket formed between two helices, h9 and h13, on the *cis*-monomer (Fig. 1 (C)).

There are three major conformational transition stages of the LTag molecular motor, which is associated with the ATP binding stage, followed by the ATP hydrolysis and the ADP releasing stages. In this report, we focus on the study of the ATP binding stage. We have built a pre-Apo state model by placing six ATP molecules 20 Å away from the nucleotide binding pocket, and an ATP docking stage model by putting the ATP in the binding pocket of the Apo state. We simulated a 1 ns (nanosecond) pathway from pre-Apo state to the Apo ATP binding states and a 1 ns pathway from pre-Apo to the ATP docking state and finally to the ATP binding state.

### LTag helicase ATP binding model

To study the LTag helicase overall conformational changes during ATP binding, we took a closer look at the ATP binding pocket by analyzing the trajectory involving the ATP ligand binding process. In this section, we studied the conformational changes of the *cis* and *trans* residues involved in ATP binding, the movement of ATP, and the dynamic hydrogen bond formation during the ATP binding process. The results of this study suggest a cross-locking model of the binding pocket for ATP binding.

#### The cross locking mechanism of the ATP binding pocket

**Figure 2:**
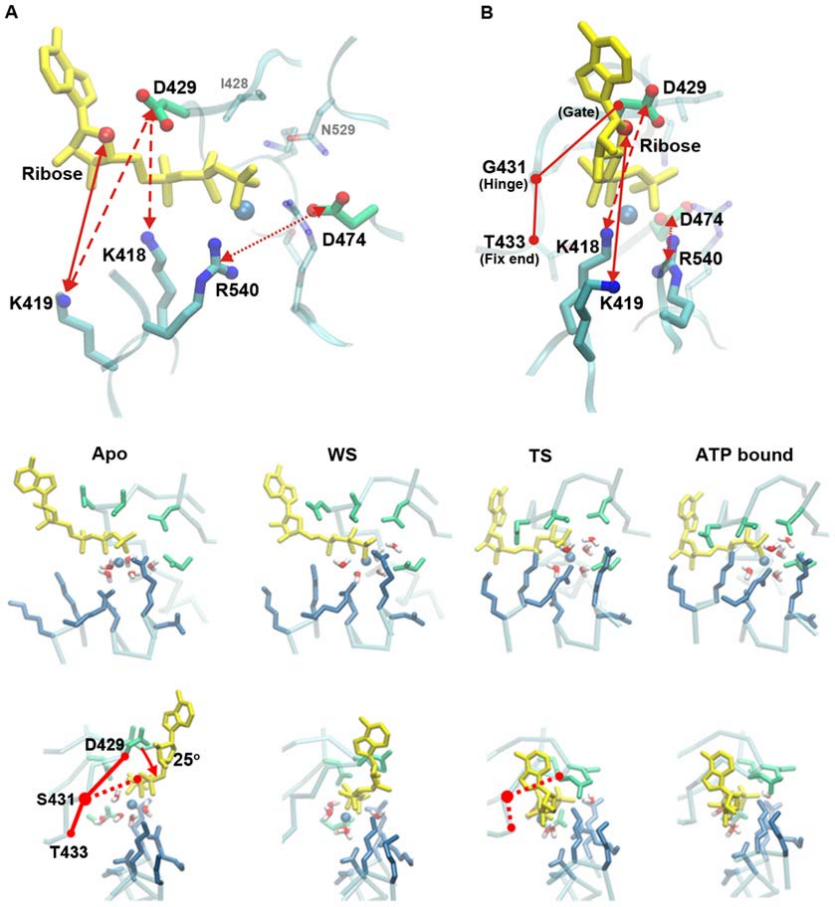
The ATP binding procedure. **(A,B)** The bottom and side views of the binding pocket residue and the ATP plus Mg^2+^ (the sphere colored in blue) complex. The residue T433 (the fixed end), G431 (the hinge) and D429 (the gate) form the ATP gate **(B)**. Lock 1 is illustrated as twin-headed arrow with solid line, Lock 2 is illustrated as dashed line and Lock 3 is represented as dotted line. The bottom two rows of figures represent the views from the bottom and the side of the ATP binding pocket, showing the four key snapshots during ATP binding process, namely the Apo state, WS, TS and ATP bound state. The oxygen atoms near the gate are illustrated as red balls in the ATP bound state.

Four key snapshots from the open state to the closed state of the simulation trajectory are illustrated in Fig. 2, namely Apo state (at the start point of the simulation), weak binding state 1 (WS, at around 0.1 ns), tight binding state (TS at around 0.4 ns) and the ATP bound state. It is important to notice that the time in the TMD simulation only represents the conformational change order, not necessarily the exact time needed for the event to occur. Similar to the F1-ATPase ATP binding model [8], the binding procedure can be divided into a docking stage from Apo to WS, and a binding transition stage leading to TS. In addition, there is a shrinking stage from TS to the ATP bound state which corresponds to the major conformational change triggered by the ATP binding [3]. For the convenience of description, we first denote the following representative measurements.

1. The *cis*- and *trans*- residues around the ATP-pocket move closer to each other during the ATP binding and form a cross locking system of three pairs of “locker” residues. The polarity of each locker residue pair is opposite to each other. As illustrated Fig. 2, we name these locks: lock1 (Ribose and LYS419), lock2 (ASP429 and LYS418/419) and lock3 (ASP474 and ARG540) (Fig. 2 (A) and (B)). Residue ASP429 blocks the ATP binding pathway after TS and acts as a gate to protect further nucleotide binding or escape. ASP429 is hinged at SER431, which is located at the relative fixed part of the P-loop with THR433. Together, ASP429-SER431-THR433 form a gate mechanism for the binding pocket (Fig. 2 (B)).
2. To study the ATP dependent conformational change, which is described as twisting and bending during ATP binding, the variance of an ATP dihedral angle (N1-C4′-C5′-PB) and an ATP angle (N1-C4′-PB) between the adenosine group, ribose and the phosphate group are traced during the binding process (Fig. 1 (C)).
3. Most of the binding procedures are described from the bottom (C-terminal) view of the helicase. We denote the positions near the central channel surface as the inner side and the positions away from the central channel as the outer side. The N terminus is labeled the upper side and the C-terminus as the bottom side.

#### ATP docking between Apo and WS

**Figure 3:**
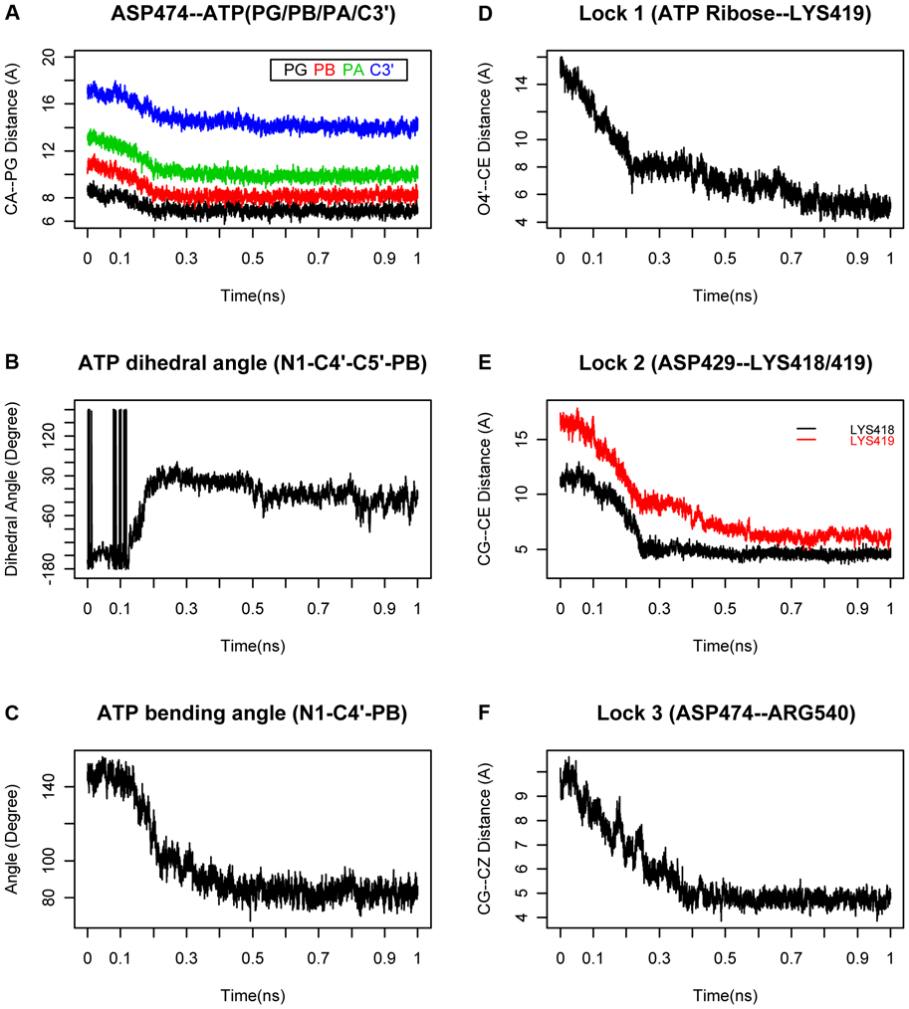
The ATP conformational change and the cross locking system in ATP binding to the pocket. **(A)** The binding direction of the ATP-Mg^2+^ complex. In the PDB file, the PA, PB and PG represent the αPi, βPi, and γPi respectively. The black, red, green and blue lines represent the distance from the αPi, βPi, γPi and ribose to the relative stable ASP474 Ca in the inner side of the binding pocket. **(B)** The dihedral angle of ATP; **(C)** The bending angle of ATP; **(D)** Lock1, the distance plot between ATP ribose O4′ and the LYS419 CE. **(E)** Lock2, the distance plot between ASP429 CG and the LYS419/419 CE. **(F)** Lock3, the distance plot between ASP474 CG and the ARG540 CZ.

The docking stage starts from the position where the ATP is around 5 Å away from the pocket. The phosphate group of ATP-Mg^2+^ complex diffuses to and docks into the binding pocket (Fig. 2 (ATP)). The positively charged residues in the binding pocket, such as LYS418, LYS423, ARG540, ARG498 begin to orient themselves to point at the negatively charged phosphate group of oncoming ATP (Fig. 2 (Apo)). The dihedral angle remains about −140 degrees (Fig. 3 (B)), the ATP angle vibrates above 130 degree (Fig. 3 (C)), and the ATP gate angle is around 140 degree, fully opened for ATP to enter the binding pocket. At the end of the diffusion, the phosphate group diffuses to the position ∼3.5 Å away from that in the final TS (Fig. 3 (A)). At this stage, the adenine is still outside the pocket, and is named as weak binding state (WS).

#### ATP binding from WS to TS

The next stage is the binding transition stage from WS to TS. The adenosine is inserted into the gap between h9 and h13 (Fig. 1 (C)), resulting in conformational changes of ATP by twisting the base into the hydrophobic pocket that accommodates the base. Our simulation reveals that the ATP finishes its major conformational change before 0.2 ns, while the cross locking residues accomplish their movement after 0.2 ns.

The ATP insertion is affected by the movement of the phosphate group to its binding position. As illustrated in Fig. 3 (A), the distances between the pocket residue ASP474 and the phosphate groups (αPi, βPi, γPi), and between ASP474 and the ribose C3′ decrease simultaneously. The relative conformations of the three phosphate groups are nearly stable. With the development of binding, the van der Waals forces accumulate between the phosphate and the binding pocket, and the forces generally prompt the adenine insertion. The adenine turns along the phosphate axis about 120 degree (Fig. 3 (B)) and finally is buried into the gap between helicase h13 and h9 (residue group ARG548 - LYS554 and SER430 - GLY431) [3]. The insertion is paired with the ATP bending. Fig. 3(C) shows that the ATP adenine group and the ribose bend about 40° toward the phosphate group, while the ATP gate closes about 25° Fig. 2 (Apo).

**Figure 4:**
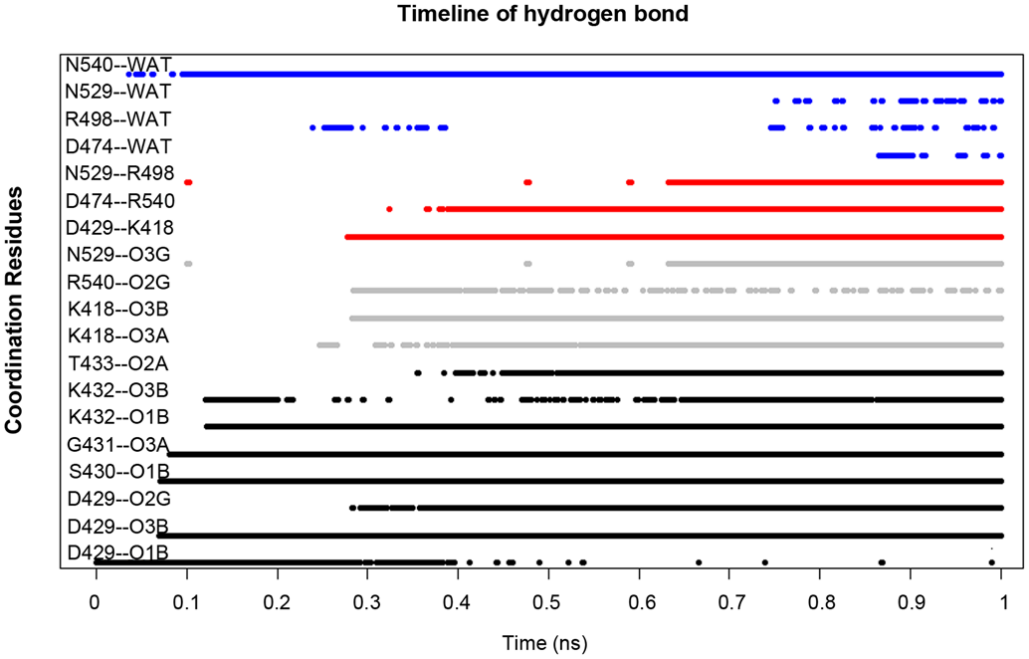
The timeline of the major hydrogen bonds formed in the binding pocket during the ATP binding procedure. The hydrogen bonds formed between the ATP and the *cis*-residues are plotted in black lines, between ATP and *trans*-residues are plotted in grey lines, between the binding lock residues are plotted in red lines, and between apical water and the coordinated residues are plotted in blue lines.

During the insertion, the residue pairs of the cross locking system also move closer to each other approaching the minimum allowed distance, which is shown by the decreasing slopes in Fig. 4D–F. At this point the “lock” is fully “locked”. From Fig. 3D–F, we can see that the three locks reach their fully locked state sequentially. The first slowdown point occurs (Fig. 3(D)) on lock1 after 0.2 ns, it is followed by the second slowdown point (Fig. 3(E)) on lock2, and then the third point on lock3 (Fig. 3(F)). The sequential locking procedures is similar to the “binding zipper” model in the F1-ATPase, where the binding affinity increases progressively [14]. At the same time, the ATP angle finishes minor adjustments to the TS position (Fig. 3(C)). The gate is fully closed to prevent the insertion of other ATP (Fig. 3 (TS)). Three residue pairs cross each other and form a cross locking system.

The mutation study [15] indicates that there are three groups of critical residues for the ATP binding: 1) the D429, S430, G431, K432, T433 on the P-loop; 2) the N529, D474 on the Sensor I motif, and 3) the arginine finger tR540 and lysine finger tK418. By analyzing the trajectory, we found that all four groups form strong hydrogen bonds with the phosphate group. The ATP interacts with P-loop residues first, and then with the rest of the residues in the binding pocket. As illustrated on Fig. 4, the hydrogen bonds are formed between the βPi group and residues D429, S430. At the same time, G431 forms hydrogen bonds with the αPi group. In the binding transition stage, the βPi group begins to further interact with the P-loop residues. For example, the new hydrogen bonds are formed between K432 and the βPi group, and then between T433 and αPi sequentially (Fig. 4). Then the lysine finger tK418 forms hydrogen bonds with the αPi and βPi groups. In addition, the arginine finger tR540 forms hydrogen bonds with the γPi group, and the Sensor I residue N529 also forms a weak hydrogen bond with the γPi group. Further, the ATP establishes coordination with the pocket residues through an apical water during binding; for example, the apical water forms hydrogen bonds with the arginine finger tR540 in the docking stage, with another arginine residue tR498 and the sensor I residue N529 in the transition stage, and with D474 in the later shrinking stage (Fig. 4, blue lines). The mutation study shows the lack of tR540, tR498, N529 and D474 causes significant reduction of ATPase activity, the formation of these hydrogen bonds might help preparing the nucleophilic attack reaction for the ATP hydrolysis later on [15].

As shown in Fig. 4, the number of hydrogen bonds increases linearly with time. The sequential formation of these hydrogen bonds ensures nearly constant force generation, which may lead to the smooth closing motion of the binding pocket throughout the whole duration of the binding transition. This observation matches the corresponding sequential binding procedure in the F1-ATPase ATP binding. The majority of the hydrogen bonds is formed between the ATP-Mg^2+^ complex and the *cis*-residues on the P-loop at the beginning. With the development of the binding procedure, the *trans*-residues, such as tR540, tK418 and tR498 form the hydrogen bonds within the binding pocket. This corresponds with the experimental results [15] that the ATP-Mg^2+^ complex attached to the *cis*-residues of the binding pocket first, and then forms the interaction with the *trans*-residues.

#### Shrinking stage from TS to ATP bound state

**Figure 5:**
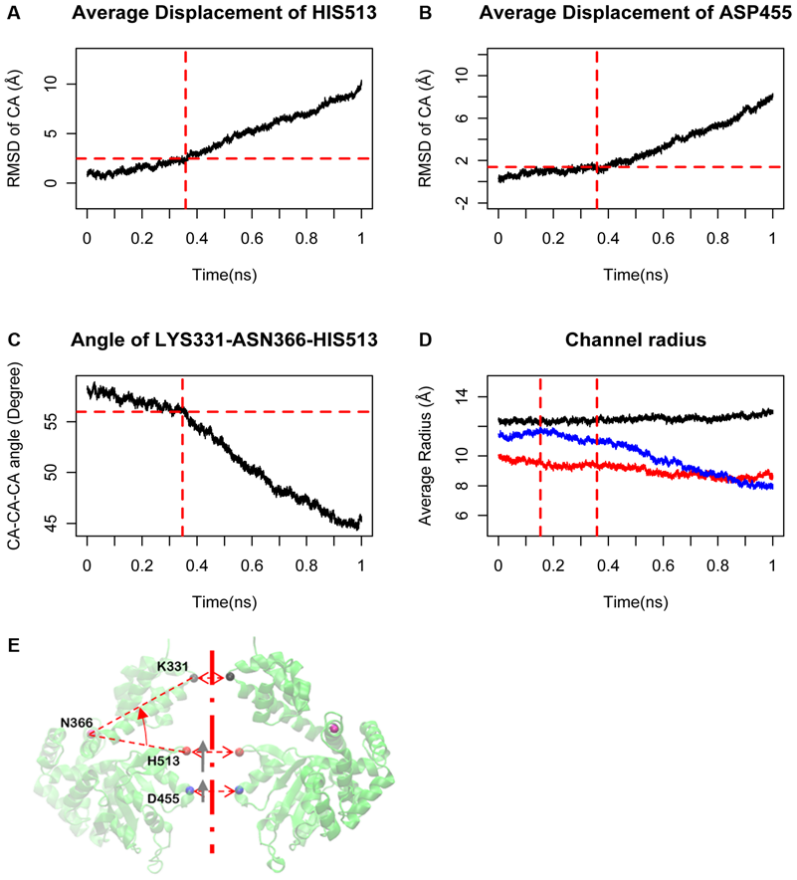
The movement at the sub-domain level. **(A)** The C-α displacement of the tip residue H513 on the β hairpin. **(B)** The C-α displacement of the tip residue D455 on the central channel of the C-terminal. **(C)** The average folding angle of D2/D3, represented by the angle between C-α of LYS331, ASN366 and HIS513. **(D)** The average channel radius on the top (black), middle (red) and bottom section (blue). **(E)** The angle that can be changed between two domains (domain folding angle) is indicated (red arrow), C-α positions of the top K331, middle H513 and bottom D455 are shown in the helicase. The H513 and D455 displacements are shown in grey arrows.

The binding of ATPs to the pocket triggers the global change after the tight binding stage: the D2/D3 domain of each monomer folds upwards to the D1 domain (Fig. 5). From the global point of view, the six domain folding movements are integrated into a shrinking movement of the C-terminal domain inwards to the central channel and upwards towards the N-terminus (Fig. 5(E)). We name this stage from TS to the ATP bound state as the shrinking stage. From Apo state to WS, to TS to ATP bound state, the cooperative conformational change of the six D2/D3 sub-domain folding movements resemble that of a closing iris, thus named the iris model [3]. During the shrinking stage, the conformations and positions of the bound ATPs remain relative stable, since all of the binding pockets are closed. The major conformational change is caused by the D2/D3 upwards movement (Fig. 5(C),(E)). It is interesting to notice that, given a fixed N terminal: 1) the central channel residues on the middle section move faster than those on the bottom 2) The residues in the bottom section move faster in the shrinking stage than in the binding transition stage. For example, residue H513 and D455 are located at the middle and bottom of the central channel respectively. The average RMSD slope of the α carbon of residue H513 is ∼12 Å/ns in the binding transition stage, and ∼14 Å/ns during the shrinking stage (Fig. 5 (A)). The average RMSD slope of α carbon of residue D455 is ∼5 Å/ns during the binding transition stage and ∼14 Å/ns during the shrinking stage (Fig. 5 (B)). These observations indicate that the β-hairpin movement is originated by the state transition of the binding pocket. After TS, the movement is mainly caused by the domain folding. That the middle section (H513) of the central channel moves faster than the bottom section (D455) may cause DNA stretching in the middle section of the central channel, which agrees with the recent crystal structure with DNA in the central channel (Data not shown).

Fig. 5(C) illustrates the change of the domain angle (LYS331-ASN366-HIS513) between D1 and D2/D3. This angle also changes at two different rates. The rate in the shrinking stage is four times faster than during the binding transition stage. Results from these figures all suggest that the major D2/D3 movement is triggered after ATP is tightly bound to the pocket, which is consistent with the observation in the literature [16].

We found that the radius of the central channel at the C-terminal end decreases faster than that in the middle section. While the central channel radius at the N-terminus remains essentially unchanged (Fig. 5 (D)). This result infers that the shrinking force comes from the D2/D3 domain, which can be used for pushing the DNA from the C-terminal to the N-terminal through the channel (Fig. 5(E)).

### Mg^2+^ coordination

**Figure 6:**
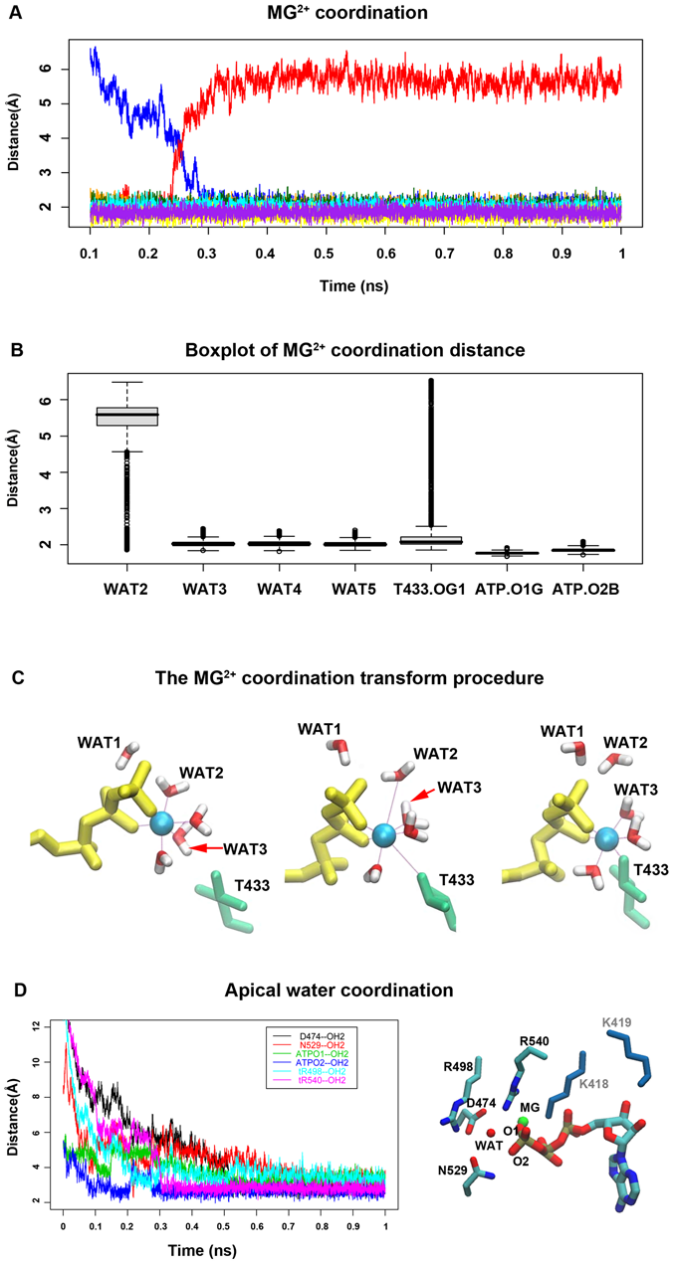
Mg^2+^ coordination transition procedure. **(A)** The distance profile between Mg2+ cation and the coordinated residues; **(B)** The box plot of the distance between Mg2+ and the coordinated residues; **(C)** The attacking of T433 to the Mg-ATP complex. Left: The initial position of T433 before invasion. Middle: T433 invasion, WAT2/WAT2 relocation. Right: The new stable structure. **(D)** The distance between the apical water and the coordinated residues, D474(black), N529(red), ATP γPi O1(green) and O2(blue), tR498(cyan) and tR540(Magenta). All the coordination distances converge to stable values after 0.6 ps on simulation time scale. These coordinations in the ATP bound state are illustrated on the right side.

The coordination of Mg^2+^ plays an important role in the ATP binding. Similar experimental study of F0F1-ATPase shows that the addition of Mg^2+^ will increase the binding affinity of the nucleotide and helps to proceed to the tight binding state [8],[17]. The binding pocket residues coordinate with the Mg^2+^ ion directly or through some intervening water molecules. Among these intervening water molecules, the apical water, WAT1, near the γPi helps to stabilize the pocket residues, and attack the γPi during hydrolysis. In our simulation, we observed the coordination of Mg^2+^ with the intervening waters during the ATP binding procedure (Fig. 6). The Mg^2+^ has a strong propensity to assume an octahedral coordination [17]. During the ATP docking stage, the Mg^2+^ ion forms a complex of six-element structure with the β, γ oxygen and four water molecules. The whole complex (ATP-Mg^2+^ and five coordinated water molecules) docks into the binding pocket until the WS. There is a flattening stage in the distance profile between *cis*-residue T433 and the Mg^2+^ (Fig. 6), which indicates that the T433 is searching for a best position to attack the Mg^2+^ in the complex. At the binding transition stage, T433 begins to attack the Mg^2+^. The invasion pushes one of the coordinated waters, WAT3, close to its neighbor WAT2, which forces WAT2 to leave the stable coordination position with the Mg^2+^ cation (Fig. 6(C)). As we can see from Fig. 6(A), there is a steep decrease in the distance between T433 and Mg^2+^ together with a sharp increase of the distance between WAT2 and Mg^2+^. On the other hand, the distance variation between WAT3 and Mg^2+^ is subtle (Fig. 6(B)). The stable coordination distance between Mg^2+^ and ligand is ∼2.0 Å. The coordination transition indicates that hydration waters may not necessarily be stripped at once. As in the case of F1-ATPase, the ATP may progressively exchange its hydrogen bonds with the hydration waters for hydrogen bonds with the ATP-pocket residues [8].

### Apical water coordination

When the ATP-Mg^2+^ complex diffuse near the pocket, the negatively charged phosphate group will interact with positively charged or polar amino acids, such as Arginine (R540,R498), Lysine(K418,K419) and Asparagine (D502,D474). However, at the beginning, these charged groups may form hydrogen bonds with waters. When the ATP-Mg complex comes in, the waters may act as temporary bridges that should be weakened and broken with molecular vibrations during ATP-Mg^2+^ binding, and eventually be replaced and expelled by the ATP-Mg^2+^ complex. However, some of these water molecules will act as bridges via hydrogen bonds between the charged amino acids and the ATP-Mg^2+^ during the entire binding process. The 2.0 Å crystal structures of LTag in different nucleotide bound states also reveal some of these fixed water molecules in the binding pocket before and after ATP-Mg^2+^ binding. Here, we focus on the apical water and the water molecules coordinated with Mg^2+^ since they are directly related to the hydrolysis of ATP. Our experimental result show that the apical water is unusually coordinated through four residues: two *cis*-residues D474, N529 and two *trans*-residues tR540 and tR498 (Fig. 6(D)). There is no particular order of coordination observed during the binding procedure. The distance between the four coordinated residues and the oxygen of the γPi group varies until the shrinking stage, when all the coordination distances converge to a stable hydrogen bond distance around 3.5 Å. The coordination procedure could be considered as a shrinking cage for the apical water (Fig. 6(D)). The vibration of the water molecule decreases until the cage shrinks to the stable state, at which point the apical water will be in a position ready for the nucleophilic attack in ATP hydrolysis.

### Interaction energy

**Figure 7:**
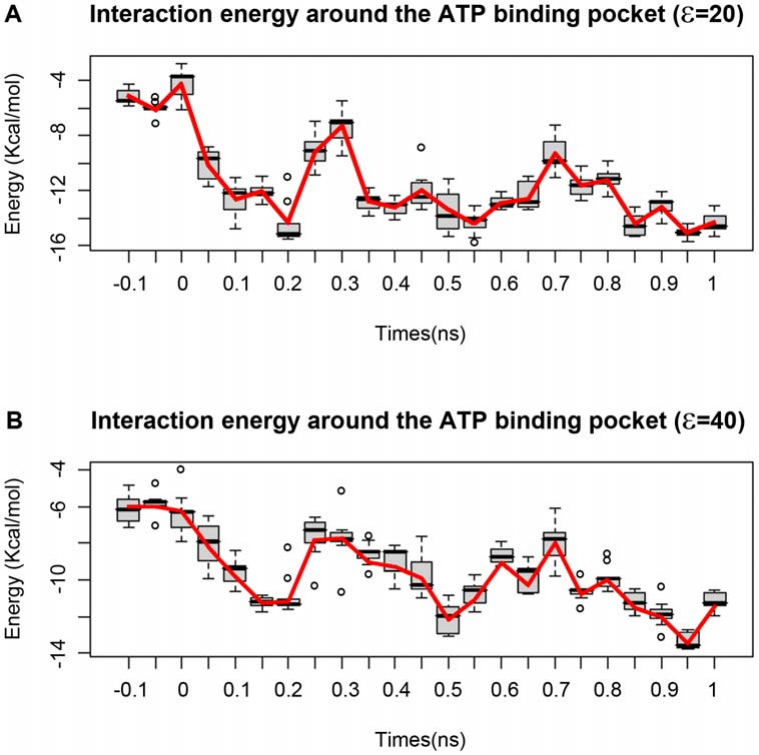
The interaction energy profile between the ATP-Mg^2+^ complex and the binding pocket. The negative time slot represents the conformation before the docking stage. The top profile **(A)** is generated with ε=20, and the bottom profile **(B)** is generated with ε=40.

The PDLD/S-LRA method is used to evaluate the binding energy of a series of 20 key snapshots (intermediate structures) sampled from the TMD simulation trajectory. The results give a rough binding pocket energy profile between the Mg-ATP complex and binding pocket. Fig. 7 (A) and (B) show results using dielectric constants of 20 and 40 respectively. The calculated trends do not depend on the choice of protein dielectric constant. The energy profile starts at −6 kcal/mol, which corresponds to the interaction between Mg-ATP complex and the water from the beginning. And we use −6 kcal/mol as a base line to measure the binding energy. There is an energy barrier of 8 kcal/mol from WS to TS (Fig. 7(A)). The time corresponds to the Mg^2+^ coordination exchange, where the WAT2 (Fig. 6(C)) escapes from its stable position due to the invasion of residue T433. The coordination transition is similar to the transition from the Mg-ATP diphosphate coordination state and Mg-ATP tri-Phosphate coordination state. In the diphosphate coordination state, the Mg^2+^ coordinates with ATP through β and γ phosphates. In the triphosphate coordination state, the Mg^2+^ coordinates with ATP through all three phosphates. The transition energy barrier is around 11 kcal/mol (18 K_b_T) in the water [18], slightly larger than our simulation results. One possible explanation is that the conformation of the binding pocket protein may facilitate the coordination transition of Mg^2+^ by decreasing the barrier about 3 kcal/mol. This is followed by an energy valley of 13 kcal/mol, which lasts throughout the binding transition stage, and ends at the beginning of the shrinking stage. Then comes another energy barrier of 5 kcal/mol. There are three hydrogen bonds formed with N529 at this time (Fig. 4). One bond is formed with the γPi group oxygen, the other is formed with the tR498 and the third is formed with the apical water. The adjustment in the shrinking stage helps to prepare the apical water to attack the γPi in the following ATP hydrolysis stage. The energy profile stablizes at −12 to −14 kcal/mol, and the binding energy is about 8 kcal/mol. The experimental result of the TNP adenine nucleotide analogues binding energy is −8 kcal/mol (−33 kJ/mol) [19], which could be used as a reference of our simulation results. Therefore, we conclude that our simulation results fall within a reasonable range, in comparison with the previous studies [8]. The major energy barrier is in the binding transition stage. Most of the binding energy is released during the docking and binding transition stage. On the other hand, most of the domain scale conformational changes happen after the binding transition stage (Fig. 5). The sequence may imply that the domain scale is triggered by the ATP binding. Similar models have been reported, for example, in the F0F1-ATPase model, the energy transduction takes places during the binding transition stage as well [8]. Some recent studies of similar T7 helicase [20],[21] also reported that the global conformational change is triggered either by ADP release or by ATP binding. The motor domain engages with DNA after ATP binding [16].

### Monomer conformational change triggered by ATP binding

**Figure 8:**
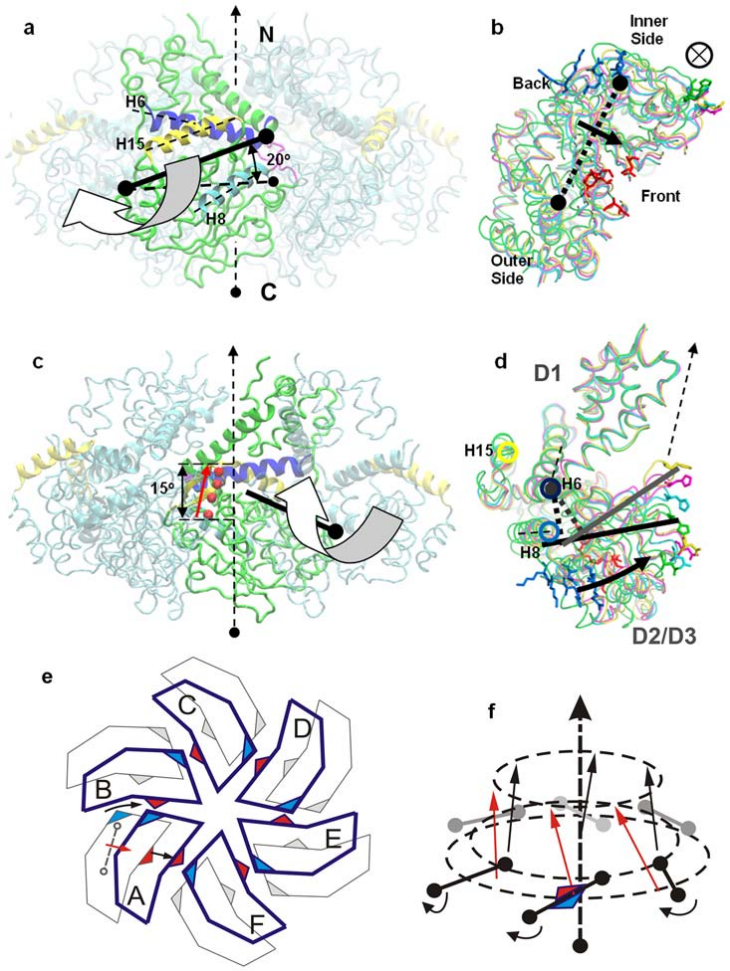
Domain-scale conformational change. **(A)** A side view of a monomer in the context of a LTag hexamer, viewing from the outside. The yellow, blue and cyan helices are the alpha-helices H15, H6 and H8 respectively. **(B)** Bottom view of the monomer. The dotted line with two round ends is the axis, along which, the D2/3 part moves around. The circle with a cross inside indicates the position of the central channel. The red and blue residues are *cis* and *trans* residues respectively. **(C)** A side view of the monomer in the context of the LTag hexamer, viewing from inside of the hexamer. The movement of the tip residue of the β hairpin (H513) is illustrated in a series of red dots. The moving trajectory is about 15° to the axis of the central channel. **(D)** Side view of the monomer perpendicular to the rotation axis. The D2/D3 movement is illustrated by a series of tip residues, such as H513 and D455. The green, cyan, yellow residues correspond to the position of WS,TS and ATP bound state. The circles in yellow, dark blue and cyan represent the axis position of H15, H6 and H8, respectively. **(E)** The cooperative iris movement of the D2/D3 domain from the bottom view. **(F)** The cooperative upwards movement of the β-hairpin along the central channel in a screw manner. The upward arrows represent the H513 movement on the tips of the β-hairpin. The curved arrows illustrate the domain folding movement of D2/D3 along the axes in solid line.

The above simulation results indicate that the domain wise conformational changes happen in the ATP binding transition and the shrinking stages. The most significant conformational change is the D2/D3 domain movement towards the D1 domain (or D2/D3 folding). The major folding movement occurs in the shrinking stage of the ATP binding process, with ∼20% occuring in the ATP binding transition stage. Our previous work has illustrated a ∼17 Å movement on the tips of the β-hairpin [3]. In this simulation study, we found that these two movements can be derived from the angled D2/D3 folding movement toward the N-terminal D1 domain, with an angle of approximately 20°. And the hinge point for the angled folding movement is around the joint of helix h5 and h6 (Fig. 8(A)). From the bottom view (Fig. 8(C)), the folding pushes the ATP-interacting *cis*-residues in an anti-clockwise direction to the neighboring *trans*-residues to form the cross-lock interactions to lock the ATP in the binding pocket. Fig. 8 (C) illustrates an interesting movement of the β-hairpin during the folding. The tip of the β-hairpin moves upward along the central channel with a screw motion, which is consistent with the simulation results in section 1.

### The cooperative movement and the iris model triggered by ATP binding

Because the LTag monomer conformational changes triggered by ATP binding occur in the context of a hexamer, the six monomers within a hexamer have to cooperate with each other during the conformational switch. Our simulation shows that the most significant cooperative movement is the formation of the ATP binding pocket and the concomitant domain-wise folding of D2/D3 in the first transition stage. The *cis*-residues for ATP binding sit in the front and face towards the folding direction. The folding movement pushes the *cis*-residues to the position with the shortest locking distance (bonding distance) with the corresponding *trans*-residues of its anti-clockwise neighbor monomer for ATP binding. At the same time, the folding movement of the neighboring monomer slides the *trans*-residues, which are located at the right side of the monomer (Fig. 8 (E), to the contacting position for the incoming *cis*-residues. At the end of the cooperative movement, the two sides of the ATP binding pocket reach the shortest bonding distance to form the cross-locking interactions for ATP binding (Fig. 8 (E)). Accompanying the folding, six β-hairpins rotate and move along their slant axes as illustrated in Fig. 8 (D) and Fig. 8 (F). The ATP binding pocket is located at the base of the β-hairpin, thus the folding movement triggered by ATP binding could be amplified through the lever effects of six β-hairpins and transferred to the tip residues. The binding of six ATPs is therefore coupled with both the screw movements of the six β-hairpins towards the N-terminal in the central channel and the collective angle folding movement of the six D2/D3 domains towards the D1 domains, like an iris of the camera (Fig. 8 (E)). However, we could not perform reliable computational analysis of the nature and extent of the cooperativity between the subunits within a hexamer at this time due to the lack of the experimental kinetics data on the cooperativity of LTag helicase.

## Discussion

ATP binding and hydrolysis by the LTag helicase motor is essential. We have performed a simulation study of the ATP binding process by LTag helicase in order to understand the energetics of ATP binding and the associated conformational changes for LTag helicase function in DNA unwinding. Based on our simulation results, we propose a cross-locking model for the ATP binding procedure for LTag helicase. The binding model can be divided into three main stages, namely, the docking stage from Apo state to the weak binding state, the binding transition stage from weak binding state to tight binding state, and the shrinking stage from the tight binding state to the ATP bound state. The first two binding stages are similar to the binding zipper model of the F1-ATPase system.

During the ATP binding process, the Mg-ATP complexes diffuse to the binding pocket in the docking stage. And the phosphate group begins to interact with the binding pocket residues, such as the P-loop, and forms the conformation of WS. In the WS conformation, the bonding interactions between the three pairs of lock residues are not formed, and the three locks are fully open and the adenine group is completely outside the pocket. WS progresses to the TS during the binding transition stage. The Mg-ATP complex progressively forms hydrogen bonds with the residues in the binding pocket through the phosphate group and the ribose. These interactions induce the conformational changes of both ATP and the lock residues around the pocket. For the ATP, as the adenine inserts into the hydrophobic gap between h9 and h13, the dihedral angle between the adenine/ribose and the phosphate group increases about 150 degrees. The ATP also bends down to a right angle. For the binding pocket, the three locks close sequentially, first lock1 (Ribose and LYS419), then lock2 (ASP429 and LYS418/419), and finally lock3 (ASP474 and ARG540).

The hydrogen bond analysis shows that the Mg-ATP complex first interacts with the P-loop and *cis*-residues, and then forms hydrogen bonds with the *trans*-residues. The major stablizing hydrogen bonds begin to form with the *cis*-residues on the P-Loop and N529, then *trans*-residues tK418 and tR540. This corresponds with the results of the mutation study [15] and the ATP binding observation from the F0F1-ATPase system [8],[14]. The number of hydrogen bonds increases linearly in the binding process, which is consistent with the results of the zipper binding model [8].

The apical water is important for the nucleophilic attack in ATP hydrolysis. Our simulation shows the position of the apical water is stabilized during the shrinking stage. The intra-ring conformational change and the relocation of residues compress the “cage” space around the apical water, and after certain adjustments, the coordinated residues are stabilized near the apical water in the ATP binding stage.

At the end of the binding transition, the gate of the ATP pocket is fully closed. Negatively charged side chains, such as ILE428, ASP429, and ribose bases, all gather outside the gate. This may help to prevent the approach and binding of the other ATP (Fig. 3 (TS)). In the binding transition stage, all the significant movement is concentrated in the binding pocket. Only ∼20% of the domain-wise conformational changes occur at this stage, which includes a subtle D2/D3 domain movement. The major domain-wise conformational changes (∼80%) is accomplished in the shrinking stage. It is interesting to note that the radius of the central channel in the C-terminal bottom portion decreases more than the middle portion during the ATP-binding triggered conformational change, which could mean that the D2/D3 upwards movement toward the N-terminal D1 domain may generate a pushing force for moving DNA through the central channel. This movement is part of the iris-motion of LTag hexamer associated with the ATP binding and hydrolysis processes.

## Methods

All the simulation is calculated using the CHARMM program package [22] and the binding energy profile is calculated by the POLARIS module of the Molaris program package [12]. The CHARMM27 all-atom force field [23] and the TIP3 water model [24] is employed. The cutoff radius for the non-bonded interactions is 14 Å. The SHAKE algorithm is adopted to fix the hydrogen bond during the simulation [25].

We have built two models for Apo state LTag helicase. The first Apo structure is built for TMD simulation of the ATP binding procedure. Six ATPs extracted from the ATP bound state are placed 20 Å away from the original Apo state helicase structure[3]. The O software program is used to adjust the ATP spatial position [26]. The ATPs are relaxed for 10 ps at 300 K. We use the Dowser program to place the internal water for the Apo structure [27]. A water sphere of 70 Å is built to wrap around the Apo structure. 36 chloride ions and 28 sodium ions are used to neutralize the system. We quenched the system for 10000 steps and then equilibrated for about 500 ps from 0 to 300 k, this is followed by another 200 ps equilibration for 300 K. The second model is built to verify the concerted model of ATP binding. The system is built by replacing one of the Apo state monomer with the corresponding ATP bound state structure. The position of the new monomer is decided by aligning the D1 domain to that of the original Apo state monomer. The system is quenched for 10000 steps and equilibrated for 500 ps from 0 to 300 k.

The ATP bound state is scanned by the Dowser program to place the missing inner water. A 70 Å TIP3 water sphere wraps the system. Again, we quench the system for 10000 steps and equilibrated it from 0 to 300 K.

The TMD simulation used an additional energy term based on the RMSD of the initial structure and final (target) structure. The energy term has the form: 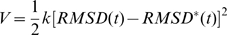, where k is the force constant (20 kcal·mol^−1^·Å^−2^), RMSD(t) is the root means square distance of the current simulated structure from the target structure, and RMSD*(t) is the predefined target RMSD value at time t. Since the forward and backward trajectory pathways are supposed to be the same, real crystal structure data provides a good starting point. Therefore, our TMD simulation started from the equilibrated ATP bound coordinates and ends at the equilibrated Apo state for 1.5 ns. The step size is 2 fs seconds. This strategy is also employed in the previous E. coli MurD study [28]. It is important to note that the Apo state monomer might not correspond to the ATP bound state monomer with the same segment name. We aligned each pair of the monomers between the Apo and the ATP bound state and save the 15 (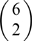) pair-wise alignment scores. Then align the six sequential monomers in the Apo state with those in the ATP bound state. For example, the segment of Apo and ATP bound state are represented by ABCDEF and A′B′C′D′E′F′ respectively. We first align the monomer sequence ABCDEF with A′B′C′D′E′F′, and then align it to B′C′D′E′F′A′, and next to C′D′E′F′A′B′, and so on so forth. The final alignment is the one with the best overall sequence alignment score. In our study, we used the last 1 ns from the trajectory. The extra 0.5 ns is removed since it is related with the surface diffusion when ATP approaching the Apo helicase, which is out of the current research.

To consolidate our results we have tried another two TMD simulations. One is the normal pathway from the equilibrated Apo state to the equilibrated ATP bound state. The conformational change is similar to the results above. Another TMD simulation involves an intermediate Apo state with ATPs bound to the pockets. The ATPs' positions are decided by aligning the ATP monomer with the Apo monomer. The intermediate Apo state is quenched and equilibrated in the same way as described above. The TMD simulation starts from the ATP bound state, and goes through the intermediate Apo state and ends at the Apo state. The simulation results are similar to the results presented above and therefore strengthen the results of our conformational pathway.

The PDLD/S-LRA (Linear Response Approximation version of the semi-microscopic PDLD) method is designed to effectively evaluate the protein-ligand binding free energies through a thermodynamic cycle that is a fast approximation of the rigorous Free Energy Perturbation (FEP) [29]. The PDLD methods have been described in a series of theoretical papers, including the PDLD method [30] , the semi-empirical version, PDLD/S [31] and the fast approximation version, PDLD/S-LRA [32] . PDLD methods have been widely applied in the related biological systems, such as the F1-ATPase [33] and HIV protease [29]. Recently, we have also successfully applied the PDLD methods on the LTag DNA translocation analysis [34].

We used the PDLD/S-LRA method to evaluate the 20 snapshots (intermediate structures) from the simulated TMD trajectory. The PDLD/S-LRA method evaluates the change in electrostatic free energies upon transfer of a given ligand (l) from water to the protein by starting with the effective PDLD potentials;
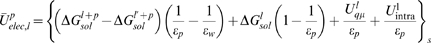
(1)

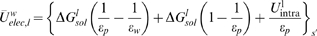
where *ΔG*_sol_ denotes the electrostatic contribution to the solvation free energy of the indicated group in water (e.g., 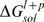 denotes the solvation of the protein-ligand complex in water). The values of the *ΔG*_sol_'s are evaluated by the Langevin dipole solvent model. 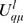 is the electrostatic interaction between the charges of the ligand and the protein dipoles in vacuum (this is a standard PDLD notation). This approach provides a reasonable approximation for the corresponding electrostatic free energies:
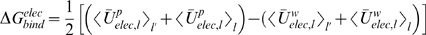
(2)where the effective potential 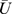 is defined in Eq. 1 and 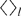 and 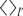 designate an MD average over the coordinates of the ligand-complex in their polar and non-polar forms. It is important to realize that the average of Eq. 2 is always done where both contributions to the relevant 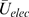 are evaluated at the same configurations. That is, the PDLD/S energies of the polar and non-polar states are evaluated at each averaging step by using the same structure.

The 20 structures are sampled evenly from the initial docking stage, through the binding transition stage (WS to TS), and the shrinking stage (TS to ATP bound state). All 20 structures are relaxed for 500 ps at 300 K. Each structure is then evaluated for 10 different runs. The mean values of these 20 structural evaluations are connected as a rough energy profile (Fig. 7).
